# Therapeutic Action of Balsam of Peru

**Published:** 1878-02

**Authors:** 


### Therapeutic Action of Balsam of Peru.

Dr. E. WISS, at a late meeting of the Medical Society of Berlin, called attention to the long-known, but lately somewhat neglected, value of the balsam of Peru in wounds, and in some other affections. He states that in all cases of cuts and lacerations, if the balsam be applied to the part injured, a momentary smart is felt, which is immediately followed by relief of the pain previously experienced, and the wound will be found to heal up with great rapidity, without suppuration, or indeed any signs of inflammation. The power of the balsam to prevent suppuration led him to employ it in chronic catarrh, as had been long ago recommended by various authors, and he mentions several cases in which the administration of it in the form of an emulsion with the yelk of egg, in the proportion of four parts of balsam to 120 of yelk, a teaspoonful being given every second hour, proved of the greatest service.—*Lancet,* Oct. 26, 1877.

